# Early mobilization for children in intensive therapy: A protocol for systematic review and meta-analysis

**DOI:** 10.1097/MD.0000000000020357

**Published:** 2020-07-24

**Authors:** Samantha Guerra Cabó Nunes Gomes, Luis Carlos Uta Nakano, Ana Carolina Pereira Nunes Pinto, Rafael Bernardes de Avila, Felipe Kenzo Yadoya Santos, Libnah Leal Areias, Virginia Fernandes Moça Trevisani, Henrique Jorge Guedes Neto, Ronald Luiz Gomes Flumignan

**Affiliations:** a Division of Evidence-based Medicine, Department of Medicine, Universidade Federal de Sao Paulo, Brazil; b Division of Vascular and Endovascular Surgery, Department of Surgery, Escola Paulista de Medicina, Universidade Federal de Sao Paulo, Brazil; c Division of Evidence-based Medicine, Department of Medicine, Universidade Federal de Sao Paulo, Brazil / Department of Physical Therapy, University of Pittsburgh, USA; d Undergraduate student of medicine, Escola Paulista de Medicina, Universidade Federal de Sao Paulo, Brazil.

**Keywords:** early ambulation, evidence-based practice, hospitalized child, rehabilitation, systematic review

## Abstract

**Introduction:**

Intensive care units focus primarily on life support and treatment of critically ill patients, but there are many survivors with complications, such as generalized muscle disorders, functional disability and reduced quality of life after hospital discharge, resulting from prolonged stays in these units. The current evidence suggests that early mobilization-based rehabilitation (exercise initiated immediately after the patient's significant physiological changes have stabilized) in critically ill adults can alleviate these complications from immobility and critical illness. However, there are a lack of practice guidelines, conflicting perceptions about safety, and knowledge gaps about benefits in the critically ill paediatric population. Therefore, we aim to assess the effects of early mobilization for children in intensive therapy.

**Methods and analysis:**

Systematic searches will be carried out in Medical Literature Analysis and Retrieval System Online, Excerpta Medica database, Cochrane Central Register of Controlled Trials, Latin American and Caribbean Centre on Health Sciences Information, Cumulative Index to Nursing & Allied Health Literature and physiotherapy evidence database databases at a minimum without date or language restrictions for relevant individual parallel, cross-over and cluster randomized controlled trials. In addition, a search will also be carried out in the World Health Organization International Clinical Trials Registry Platform, and in the clinical trial registries of ClinicalTrials.gov, looking for any on-going randomised controlled trials that compare early mobilization with any other type of intervention. Two review authors will independently perform data extraction and quality assessments of data from included studies, and any disagreements will be resolved by discussion or by arbitration. The primary outcomes will be mortality and adverse events. Secondary outcomes will include duration of critical care (days), duration of mechanical ventilation support, muscle strength, pain and neuropsychomotor development. The Cochrane handbook will be used for guidance. If the results are not appropriate for a meta-analysis in RevMan 5 software (e.g., if the data have considerable heterogeneity and are drawn from different comparisons), a descriptive analysis will be performed.

**Ethics and dissemination:**

This protocol was prospectively registered at Open Science Framework and approved by the Ethics and Research Committee of the Federal University of Sao Paulo (8543210519). We intend to update the public registry used in this review, report any important protocol amendments and publish the results in a widely accessible journal.

**Registration:**

osf.io/ebju9.

## 1 Introduction

Intensive care units (ICUs) emerged with the need to offer special care to critically ill patients, focusing primarily on life support and treatment of patients with clinical instability.^[1]^ There are many types of classifications for ICUs, and paediatric ICUs have very particular characteristics.^[2]^

Mortality rates in paediatric ICUs can vary widely (from 3% to 30%), but in the last decades, with technological and scientific advances, there has been a significant reduction in the mortality of ICU patients.^[1,2]^

At the same time, there has been a considerable increase in the number of survivors with complications resulting from prolonged stays in these units. Generalized muscle disorders, functional disability and reduced quality of life after hospital discharge are some examples of these complications.^[3]^

This is because it is common for ICU patients to remain bedridden for prolonged periods, leading to important complications resulting from immobility. Prolonged rest associated with the critically ill patient leads to decreased muscle protein synthesis, increased urine output, nitrogen excretion (muscle catabolism) and decreased muscle mass. Loss of mass and, consequently, muscle strength are the initial factors that culminate later with the development of polyneuropathy and/or myopathy of the critically ill, leading to a 2 to 5-fold increase in hospital length of stay and increased care costs.^[4]^

In paediatric ICUs, the scenario is no different, most children who survive intensive care in paediatric ICUs are more prone to recurrent infections, cardiorespiratory deconditioning, delayed neuropsychomotor development, and reduced quality of life after hospital discharge.^[5,6]^

Current evidence suggests that early mobilization-based rehabilitation in critically ill adults can alleviate complications from immobility and critical illness.^[7]^ Early mobilization, while feasible and safe, can reduce the risk of delirium, improve functional recovery, and improve outcomes, as well as reduce the use of resources in adults in the ICU.^[8,9]^

Early mobilization is defined as exercise initiated immediately after the patient's significant physiological changes have stabilized.^[10]^ In general, the exercises can be classified as: passive exercise, where there is no muscle contraction and the movements are performed by the therapist; assisted active exercise, where there is muscle contraction, but the patient needs the help of the therapist to complete the execution; active exercise, where there is muscle contraction and the patient can perform all movements without assistance but under the supervision of the therapist; and resistance active exercise, where the therapist exerts a resistance to the execution of the movement in order to enhance the quality of muscle contraction.^[11]^

Multiple barriers are cited as being related to the implementation of early mobilization in paediatric ICUs, such as lack of practice guidelines, the need for medical orders, conflicting perceptions about safety, and knowledge gaps about benefits in the critically ill paediatric population.^[12]^ Because of this, it is not clear whether this is an effective and safe intervention for the paediatric population.^[13]^ Therefore, we aim to assess the effects of early mobilization for children in intensive therapy.

## 2 Method and materials

This systematic review (SR) protocol has been prepared using and following the recommendations guidelines of the Cochrane Handbook for SR of Interventions and is reported according to the Preferred reporting items for SR and meta-analysis protocols recomendations.^[14,15]^ The current SR protocol is prospectively registered in Open Science Framework and approved by the Ethics and Research Committee of the Federal University of Sao Paulo (8543210519).^[16]^

### 2.1 Eligibility criteria

We will include individual parallel, cross-over and cluster randomized controlled trials (RCTs), which have evaluated any type of early mobilization intervention for paediatric patients in critical care. We will include studies reported as full text, published as abstract only and unpublished data. We will not consider quasi-RCTs (ie, those studies in which participants are allocated to intervention groups based on methods that are not truly random, such as the hospital number or date of birth, for inclusion.

### 2.2 Types of participants

We will include paediatric participants (ie, those from 1 month to 18 years of age and under critical care).^[17]^ We define participants under critical care as critically ill patients who require continuous specialized professional attention, monitoring and therapy.^[18]^ If we find studies with mixed populations, and only a subset of the participants meet our inclusion criteria, we will attempt to obtain data for the subgroup of interest from the trialists.

### 2.3 Types of interventions

#### 2.3.1 Intervention

The intervention performed with the critically ill patient will consist of any type of exercise started as soon as hemodynamic stabilization compared with the control group.

We will consider the following types of mobilization, combined or not:

(1) Exercises with devices such as an ergometer cycle;
(2) Active range of motion exercises;
(3) Bed mobility activities (eg, bridging, rolling, and lying to sitting);
(4) Exercises related to increasing independence with functional tasks;
(5) Ambulation;
(6) Any other type of passive or active exercise modality that commenced while the participant was in critical care.

#### 2.3.2 Comparators

The comparators may consist of:

(1) Early mobilization versus delayed intervention (mobilization/active exercise the same as the intervention group, but given later, either in critical care, or after the participant left the critical care unit);
(2) Early mobilization versus usual care (no mobilization or exercise while in critical care);
(3) Early mobilization versus respiratory muscle training only.

### 2.4 Types of outcome measures

#### 2.4.1 Primary outcomes

(1) All-cause mortality;
(2) Adverse events (eg, accidental extubation, pulmonary embolism, fall, catheter loss, surgical wound infection, irritability, delirium, nausea, vomiting, and pressure ulcer). All adverse events will be reported separately, if possible.

#### 2.4.2 Secondary outcomes

(1) Duration of critical care (days);
(2) Duration of mechanical ventilation support;
(3) Muscle strength assessed by any objective method as manual or digital dynamometer or measured with the Medical Research Council scale^[19]^ or any other validated score or scale;
(4) Pain, assessed by the visual analogue scale or any other validated method;
(5) Neuropsychomotor development assessed by any validated method, such as the Bayley Scale of Infant Development.^[20]^

### 2.5 Search methods for identification of studies

#### 2.5.1 Electronic searches

We aim to identify all relevant RCTs regardless of language or publication status (published, unpublished, in press or in progress).

We will search the following databases for relevant trials:

(1) The Cochrane Central Register of Controlled Trials via the Cochrane Register of Studies Online (CRSO);
(2) Medical Literature Analysis and Retrieval System Online via PubMed.gov;
(3) Excerpta Medica dataBASE via Elsevier;
(4) Latin American and Caribbean Health Science Information (via Virtual Health Library) database;
(5) Physiotherapy Evidence Database;
(6) Cumulative Index to Nursing and Allied Health Literature via Ebsco.

The Medical Literature Analysis and Retrieval System Online search strategy is provided in Table 1, and it will be used as the basis for search strategies for the other databases listed.

**Table 1:**
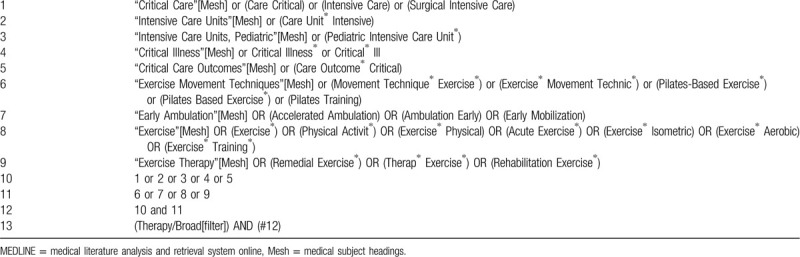
MEDLINE via PubMed search strategy.

We will also search the following trial registries for ongoing clinical trials:

(1) The World Health Organization International Clinical Trials Registry Platform (who.int/trialsearch);
(2) ClinicalTrials.gov (clinicaltrials.gov).

#### 2.5.2 Manual searches

We will check reference lists of all the included studies and any relevant SRs identified for additional references to relevant trials. We will also contact the authors of the included trials for any possible unpublished data. Furthermore, we will contact specialists in the field to enquire about relevant ongoing or unpublished studies.

#### 2.5.3 Selection of studies and data extraction

With aid of the Rayyan tool, we will examine the titles and abstracts to select potentially relevant studies after the search results are merged and duplicate records removed.^[21]^ Two review authors (SGCNG and ACPNP) will independently evaluate the studies identified by the literature search and code them as ’retrieve’ (eligible or potentially eligible/unclear) or ’do not retrieve’ (not relevant). Any disagreements will be solved by a team discussion among the authors or by arbitration with a third author (RLGF). We will retrieve the full text of the relevant trials, and 2 review authors (SGCNG and ACPNP) will independently examine and identify studies for inclusion. If a trial does not meet the eligibility criteria, we will identify and document the reasons for exclusion. If there is disagreement about the relevance of a study, we will consult a third review author (RLGF) or solve by discussion. We will illustrate the study selection process in a Preferred Reporting Items for SRs and Meta-Analyses diagram.^[22]^ We will list all articles excluded after the full-text assessment in the ’Characteristics of excluded studies’ table and will provide the reasons for their exclusion.

The following data on the study characteristics and outcomes of the included studies will be extracted by 2 independent reviewers (SGCNG and ACPNP)^[23]^:

(1) Methods: study design, total duration of the study and period of carryout, number and location of study centres, research setting, withdrawals and date of study.
(2) Participants: number, age parameters (ie, mean, range and so on), gender, the severity of the condition, diagnostic criteria and inclusion/exclusion criteria.
(3) Interventions: type of intervention, comparison, concomitant interventions, and excluded interventions.
(4) Outcomes: primary and secondary outcomes (the final outcomes reported and those planned) and time points reported.
(5) Notes: funding for trial and notable conflicts of interest of trial authors.

One author (SGCNG) will enter these data into Review Manager 5 software (RevMan 5), version 5.3, for statistical analysis.^[23]^ If the results are not appropriate for a meta-analysis, a descriptive analysis will be performed.

#### 2.5.4 Assessment of risk of bias in included studies

The critical evaluation of the included articles will be done in a double and independent way, regarding the risk of bias of the included studies. We will assess the following risk of bias domains:

(1) random sequence generation,
(2) allocation concealment,
(3) blinding of participants and personnel,
(4) blinding of outcome assessment,
(5) incomplete outcome data,
(6) selective outcome reporting and
(7) other bias, as recommended by The Cochrane Handbook.^[24]^

Each of these domains will be graded as having high, low or unclear risk of bias. Blinding will be considered separately for different key outcomes when necessary.

#### 2.5.5 Measures of treatment effect

We will perform heterogeneity analysis to evaluate the possibility of grouping the data. When at least 2 studies are sufficiently homogeneous in terms of participants, interventions and outcome measurements, we will pool their results into a meta-analysis. Meta-analysis will be performed using an inverse variance method and random effects model in Revman 5.^[22]^ Dichotomous data will be treated through relative risk, with confidence interval 95%; continuous data will be treated through the mean difference for equal scales or standardized mean difference for different scales, also with confidence intervals 95%.

#### 2.5.6 Unit of analysis issues

The participant will be the unit of analysis for all outcomes. The intention-to-treat approach will be used.

#### 2.5.7 Addressing missing data

We will contact the authors or sponsors of the included studies to request missing numerical outcome data and to verify details about methods and characteristics. If the missing data have different implications in the compared groups, the studies will be considered to have a high risk of bias. The frequency (or risk) of outcomes has a direct influence on the potential impact of missing data in dichotomous outcomes studies, while the proportion of participants with missing data is directly related to the potential impact on continuous outcomes studies.^[23]^

#### 2.5.8 Assessment of heterogeneity

Heterogeneity between studies will be analysed by visual inspection of forest plots associated with the use of the *I*^2^ consistency test. The degree of heterogeneity should not be strictly, but we will use *I*^2^ according to the guide for interpretation in the Cochrane handbook for SRs of interventions^[25]^:

(1) 0% to 40%: possibly not important;
(2) 30% to 60%: may represent moderate heterogeneity;
(3) 50% to 90%: may represent substantial heterogeneity;
(4) 75% to 100%: considerable heterogeneity.

When *I*^2^ lay in an area of overlap between 2 categories (eg, between 50% and 60%), we will consider differences in participants and interventions among the trials contributing data to the analysis.^[25]^

Data will be analysed using the random effect model. We will investigate sources of heterogeneity by subgroup and/or sensitivity analysis. Subgroup analysis is foreseen considering the age of the children, reason for hospitalization, associated comorbidities or differences related to the applied intervention. If more than 10 studies are included in a meta-analysis, we will perform publication bias analysis. Data from all trials will be compiled and analysed using the RevMan 5.^[26]^

#### 2.5.9 Assessment of reporting biases

The presence of publication bias and other types of reporting bias will be assessed using funnel plots if there are at least 10 studies for inclusion in a meta-analysis.

#### 2.5.10 Subgroup analysis and investigation of heterogeneity

We will consider all types of early mobilization in this review. In the case of substantial heterogeneity, we will do a subgroup analysis for the following characteristics:

(1) Age (eg, infants 0–2 years old, children 3–12 years old, and young people 13–18 years old);
(2) Reason for hospitalization;
(3) Associated comorbidities;
(4) Differences related to the intervention applied;
(5) Mechanical ventilation and non-invasive ventilation separately.

We will attempt to contact the authors to obtain missing data when data are not available from the original publications. We will also explore the following data: according to risk of bias and use formal testing for subgroup differences in RevMan 5 and base our interpretation on that.^[24,26]^

### 2.6 Sensitivity analysis

A sensitivity analysis will be conducted to determine the impact of exclusion studies with an overall high risk of bias, which are those studies with a high risk of bias in at least 1 of the main domains in the risk of bias tool analysing generation of randomisation sequence, allocation concealment and blinding.^[25]^

### 2.7 ‘Summary of findings’ table

To generate a ‘Summary of findings’ table for each 1 of the outcomes to be analysed in this review, we will use the Grading of Recommendations Assessment, Development and Evaluation (GRADE) software (GRADEpro).^[27]^ Using the criteria study limitations, consistency of effect, imprecision, indirectness and publication bias, we will assess the certainty of the body of evidence that made up the data for the meta-analyses of the pre-specified outcomes.^[27,28]^ These criteria will be evaluated, and the table will be filled in by using the Cochrane recommendations, justifying any departures from the standard methods.^[23]^

### 2.8 Patient and public involvement

The research question was developed from the authors’ experience treating paediatric patients that were critically ill, associated with methodological knowledge and under the advisement of a patient group for the selection of the main patient-relevant outcomes. We intend to include patients in all steps of this research as advisors and to maintain comprehensive language in the final text that will be appropriate for consumers. The final version of this review, with results, conclusions and any changes in the protocol, will be published in an accessible international journal.

### 2.9 Ethics and dissemination

The study has been approved by the Ethics and Research Committee of the Federal University of Sao Paulo (8543210519). It is hoped that the authors of primary studies to be included in the analysis have already obtained such approval. We intend to update the public registry with this review at Open Science Framework (osf.io/ebju9) in all phases of its execution, report any important protocol amendments and publish the results in a widely accessible journal.

## 3 Discussion

Our review will evaluate evidence of the efficacy and safety of early mobilization for paediatric ICU patients. The results of our SR will be of interest to managers and paediatric intensive care professionals worldwide. The information gathered in the implementation processes will inform patients, families and health professionals about their effectiveness and safety, helping to facilitate decision-making for the implementation of the practice in ICUs. This study will also identify gaps for future research.

### 3.1 Strengths and limitations of this study

(1) This SR will evaluate the effectiveness and safety of applying early mobilization in critical care paediatric patients. The method of this review includes explicit eligibility criteria, extensive database search, and independent and paired evaluations for study selection.
(2) We will assess the risk of bias in the qualitative and quantitative studies that will be included, and we will use the GRADE approach for the final evidence.
(3) Since we plan to include only RCTs, a lack of available evidence to evaluate and synthesize may be a limitation.
(4) Outcomes can guide patients, family members and intensive care professionals about the effectiveness and safety of early mobilization, helping to facilitate decision-making in the ICU setting.

## Acknowledgments

The authors would like to thank the patient advisers, Cochrane Brazil, and the Division of Vascular and Endovascular Surgery, Universidade Federal de Sao Paulo, Brazil, for their support.

## Author contributions

SGCNG: coordinated contributions from co-authors, drafted the clinical sections, contributed to writing the protocol, will perform future review updates and is the guarantor of the review. LCUN, ACPNP, RBA, FKYS, LLA, HJGN, and VFMT: worked on the methods sections and contributed to writing the protocol. RLGF: worked on the clinical and methods sections, contributed to writing the protocol and will perform future review updates.
